# The hindbrain‐hypothalamic noradrenergic pathway controls homeostatic food intake but not cue‐induced appetitive behaviors

**DOI:** 10.1111/jne.70229

**Published:** 2026-07-08

**Authors:** Colin Vuong, Jo Ann Yap, Helen Yi Sha Gu, Hun Yee Lai, Zhi Yi Ong

**Affiliations:** ^1^ School of Psychology University of New South Wales, UNSW Sydney Australia

**Keywords:** homeostatic feeding, lateral hypothalamus, non‐homeostatic feeding, noradrenergic neurons, nucleus of the solitary tract

## Abstract

Nucleus of the solitary tract (NTS) A2 noradrenergic neurons are critical in regulating food intake. In addition to their well‐established role in homeostatic feeding, there is also evidence indicating their contribution in non‐homeostatic feeding behaviors. However, whether their control of homeostatic and non‐homeostatic feeding behaviors is mediated via shared or separate neural circuits is not well understood. Given that NTS A2 neurons project to the lateral hypothalamus (LH), a brain region implicated in both homeostatic and non‐homeostatic feeding, this study examined whether NTS A2 neurons project to LH (A2^NTS→LH^) to control these feeding behaviors. We used chemogenetics to activate A2^NTS→LH^ neurons. To assess homeostatic feeding, we measured chow intake in the home cage for 24 h and to assess non‐homeostatic feeding, we used the Pavlovian appetitive conditioning task to measure cue‐induced appetitive behavior. Using anatomical tracing, we first showed that ~20% of NTS A2 neurons project to the LH and these LH‐projecting NTS A2 neurons are activated by food intake. Chemogenetic activation of A2^NTS→LH^ neurons significantly suppressed home cage chow intake and body weight. However, while activation of all NTS A2 neurons suppressed cue‐induced appetitive behavior, pathway specific stimulation of A2^NTS→LH^ neurons did not affect this cue‐driven behavior. Together, these results indicate that the A2^NTS→LH^ neurons are involved in the control of homeostatic food intake but not cue‐induced appetitive behaviors, and suggest the contribution of distinct NTS A2 pathways and LH neurons in the regulation of homeostatic and non‐homeostatic feeding behaviors.

## 1 INTRODUCTION

Food intake is regulated by internal physiological and external environmental factors.^1^, ^2^ While different neural substrates have been implicated in mediating these behaviors, recent evidence suggests that the underlying neural circuits are more neuroanatomically distributed than previously appreciated. The nucleus of the solitary tract (NTS), a key hindbrain region established for its role in regulating homeostatic food intake, has also been implicated in non‐homeostatic feeding behaviors. Specifically, leptin, glucagon‐like peptide‐1 (GLP‐1), and oxytocin receptor signaling in the NTS not only suppress food intake but also reduce feeding behaviors beyond homeostatic need, including palatable food‐seeking and food‐motivated behaviors.^3^, ^4^, ^5^ However, the specific neuronal phenotypes and circuits mediating these effects remain to be fully understood.

Our previous work, along with that of others, showed that activation of NTS noradrenergic A2 neurons suppresses food intake, irrespective of energy status or food palatability, which indicates a role for these neurons in regulating both homeostatic and non‐homeostatic feeding.^6^, ^7^, ^8^, ^9^ More recently, we showed that noradrenaline release in the nucleus accumbens (NAc), which originates from NTS A2 neurons, is attenuated following presentation of a food cue and cue‐induced approach behavior, further implicating these neurons in cue‐mediated feeding behaviors.^10^ However, whether the same or different neural mechanisms mediate the effects of NTS A2 neurons on homeostatic and non‐homeostatic feeding behaviors is unknown, which requires further investigation of their downstream projections.

Anatomical studies show that NTS noradrenergic neurons project to other brain regions involved in food intake control and reward processing, including the bed nucleus of the stria terminalis, the paraventricular nucleus of the hypothalamus (PVH), and the lateral hypothalamus (LH).^6^, ^11^ The A2^NTS→LH^ projection is of particular interest because the LH is known to regulate both homeostatic and non‐homeostatic feeding behaviors. For example, ghrelin administration to the LH increases food intake while GLP‐1 or leptin administration reduces food intake.^12^, ^13^, ^14^ Furthermore, activation of LH GABA neurons increases food intake while LH glutamatergic neurons decrease food intake.^15^, ^16^, ^17^, ^18^ LH GABA neurons are also involved in non‐homeostatic cue‐induced feeding behaviors where inhibiting these neurons disrupts associative learning and decreases cue‐induced food‐seeking behaviors.^19^ Furthermore, studies show that LH orexin neurons are activated by external cues that predict food reward and this activation is correlated with anticipation of food rewards suggesting their involvement in cue‐induced feeding behaviors.^20^, ^21^ Together, these studies indicate that LH neurons affect both homeostatic and non‐homeostatic feeding behaviors, but whether NTS A2 inputs to the LH contribute to these feeding behaviors is unknown.

This study therefore aimed to examine the role of the A2^NTS→LH^ pathway in the regulation of homeostatic food intake and non‐homeostatic cue‐induced feeding behaviors. Using anatomical tracing and chemogenetic approaches in TH Cre rats, we showed that NTS A2 neurons projecting to the LH are activated by food intake and stimulation of A2^NTS→LH^ neurons reduced home cage chow intake and body weight, without any effects on cue‐induced appetitive behaviors. These findings indicate that distinct NTS A2 circuits contribute to homeostatic and non‐homeostatic feeding behaviors.

## 2 MATERIALS AND METHODS

### 2.1 Animals

Male Sprague–Dawley TH Cre (SD‐TH‐Cre^tm1sage^, Sage Laboratories, Cambridge, United Kingdom) and wild type Sprague Dawley rats (Ozgene, Western Australia) were housed in a climate‐controlled room on a 12 h:12 h light/dark cycle (lights off at 7 pm). Rats had ad libitum access to standard rat chow (Specialty Feeds, Western Australia) and water, and were group‐housed unless otherwise noted. The number of rats in each group was determined based on previous published work.^3^, ^6^, ^10^, ^22^ All experiments were approved by the University of New South Wales Animal Ethics Committee.

### 2.2 Surgery

Rats were anaesthetized intraperitoneally (i.p.) with a mixture of Xylazine (0.3 mL/kg; Rompun:Bayer) and Ketamine (100 mg/mL; Ketapex, Apex Laboratories), and received subcutaneous injections of bupivacaine, a local anaesthetic, near the incision site. For anatomical tracing experiments, wild type Sprague Dawley rats received infusions (100 nL) of a retrograde tracer, Fluorogold (FG; Fluorochrome Cat# Fluoro‐gold, RRID: AB_2314408) to the LH (Bregma −2.5 mm, Midline ±2 mm, Skull −8.88 mm).^23^ For NTS Designer Receptors Exclusively Activated by Designer Drugs (DREADD) experiments, TH Cre rats were used to target and manipulate NTS A2 neurons, as previously validated.^6^ A cre‐dependent AAV encoding the excitatory DREADD hM3Dq (pAAV‐hSyn‐DIO‐hM3Dq) or reporter control enhanced green fluorescent protein (pAAV‐hSyn‐DIO‐eGFP) or mCherry (pAAV‐hSyn‐DIO‐mCherry) was infused (200 nL/side) into the NTS (Occipital +1.9 mm, Midline ±0.5 mm, Skull −9.3 mm [15° anterior–posterior]).^23^ All infusions were made at a rate of 100 nL/min and the injector was left in place for a further 5 min to ensure complete diffusion. The wound was then sutured. For A2^NTS→LH^ DREADD experiments, in addition to NTS infusions of either pAAV‐hSyn‐DIO‐hM3Dq or pAAV‐hSyn‐DIO‐eGFP or pAAV‐hSyn‐DIO‐mCherry, rats were also implanted with a bilateral guide cannula targeting the LH (Bregma −2.5 mm, Midline ±2.0 mm, Skull −8.6 mm).^23^ This enables CNO delivery to the projection target to modulate synaptic signaling of a defined neural pathway (i.e., A2^NTS→LH^ neurons), as previously shown.^6^, ^24^, ^25^, ^26^, ^27^ An injector tip extending 1 mm below the cannula was used during microinjections. The cannula was secured to the skull with 3 jeweler screws and dental cement. A dummy cannula tip was inserted into the cannula, covered with a cap to prevent any blockage in the cannula. After surgery, rats were allowed to recover for a week. Rats in the homeostatic home cage food intake experiment were individually housed after surgery.

### 2.3 Viral vectors

pAAV‐hSyn‐DIO‐mCherry was a gift from Bryan Roth (Addgene viral prep #50459‐AAV5; http://n2t.net/addgene:50459; RRID: Addgene_50459), pAAV‐hSyn‐DIO‐hM3Dq was a gift from Bryan Roth (Addgene viral prep #44361‐AAV5; http://n2t.net/addgene:44361; RRID: Addgene_44361), pAAV‐hSyn‐DIO‐eGFP was a gift from Bryan Roth (Addgene viral prep #50457‐AAV5; http://n2t.net/addgene:50457; RRID: Addgene_50457).

### 2.4 Deprivation re‐feed

Five days after receiving FG infusions to the LH, all wild‐type Sprague Dawley rats were food deprived for 24 h. Rats were randomly allocated to Deprived (*n* = 3) or Re‐fed (*n* = 3) groups. Deprived rats remained food deprived while rats in the Re‐fed group were given ad libitum access to chow and allowed to consume for 2 h prior to perfusions. Average food intake per rat during the 2 h period was 10.6 g.

### 2.5 Home cage chow intake

TH Cre rats (hM3Dq *n* = 7, eGFP *n* = 6) were acclimatized to individual housing and microinjections one week before commencement of experiments. During test days, food was removed 2 h before dark cycle onset to ensure similar energy status between rats. Forty‐five minutes prior to dark cycle onset, rats received 0.5 μL/side bilateral LH microinjections of CNO (1.5 mM, 3 mM; dissolved in 5% Dimethyl‐Sulfate Oxide [DMSO] and 95% saline) or vehicle (VEH), delivered at a rate of 1 μL/min. Chow was returned at dark cycle onset and food intake was measured after 2, 16 and 24 h. Body weight was also measured before injections and at 24 h.

### 2.6 Cue‐induced appetitive behavior

Pavlovian appetitive conditioning task was used to measure cue‐induced appetitive behavior. The task was conducted in standard Med Associates rat conditioning chambers. TH Cre rats were trained to associate an auditory cue of either a clicker or a tone with the delivery of a sucrose pellet (CS+) or no reward (CS−). The auditory cues were counterbalanced such that one group of rats received the tone as the CS+ and the clicker as the CS−, while the other group received the reverse. Each session consisted of a randomized order of 8 CS+ and 8 CS−, with each CS lasting 15 s with an inter‐trial interval of 4 min. Elevation scores were calculated based on the difference of the number of magazine entries 15 s before CS± onset from magazine entries made during the 15 s of CS±.

To examine the role of the A2^NTS→LH^ pathway on cue‐induced appetitive behavior, during test days, TH Cre rats (hM3Dq *n* = 8, mCherry *n* = 4, eGFP *n* = 3) received bilateral LH microinjections of either 0.5 μL 3 mM CNO or 0.5 μL VEH delivered at a rate of 1 μL/min. After 45 min, they were placed into operant chambers and completed a Pavlovian appetitive conditioning session. mCherry and eGFP were used as reporter controls across different experimental batches. Data from rats expressing mCherry and eGFP were combined for analysis because they were subjected to the same experimental conditions and showed no differences in elevation scores during VEH treatment (CS+: *t*_5_ = 0.714, *p* = .507; CS−: *t*_5_ = 0.173, *p* = .869). While formal statistical equivalence could not be established given the small sample size in each group, the lack of differences in experimental conditions and behavior provided a reasonable basis to combine the groups.

To confirm that activation of NTS A2 neurons suppresses cue‐induced appetitive behavior, on separate days, a subset of these rats (*n* = 10; hM3Dq *n* = 6, mCherry *n* = 4) also received i.p. injections of either VEH or 1 mg/kg CNO, 45 min prior to test session commencement. All treatments were counterbalanced and separated by at least 48 h to prevent any drug carry‐over effects.

### 2.7 Transcardiac perfusions

Rats were perfused 1 week after completion of behavioral experiments or 2 h after access to chow for the anatomical tracing experiment. Rats were injected with lethabarb (diluted 1:1 in saline), and transcardial perfusion was conducted using prewash (dH2O with 0.9% NaCl, 1% sodium nitrate, 5000 iu/ml heparin) followed by 4% paraformaldehyde. Brains were extracted, post‐fixed in paraformaldehyde overnight, and then transferred into 20% sucrose solution for cryoprotection. Brains were stored at 4°C until ready for slicing.

### 2.8 Tracer, viral and cannula placement verification

To confirm FG placement at the LH, 35 μm LH sections were obtained and mounted onto slides. Slides were then coverslipped with Fluoromount™ Aqueous Mounting Medium (Sigma Aldrich, United States) and visualized under the UV filter of a fluorescence microscope. The presence of FG in the LH indicated accurate targeting.

To confirm LH cannula placements, rats received microinfusions of 0.2 μL 1% Pontamine Sky Blue (Sigma Aldrich, United States) to the LH prior to perfusions. LH sections were collected and dry mounted on slides. The sections were then visualized, and images were taken using a brightfield microscope at 4X magnification.

To verify viral expression, 35 μm NTS sections were collected, and immunofluorescence (see below) was performed before they were mounted onto slides and coverslipped with Fluoromount™ Aqueous Mounting Medium (Sigma Aldrich, United States). Rats with off‐target placements were excluded from the analysis (*n* = 6 no hM3Dq expression, *n* = 5 no eGFP expression, *n* = 10 misplaced cannula).

### 2.9 Immunofluorescence

NTS sections were sliced at 35 μm and washed in 0.1 M phosphate buffer saline (PBS) before incubating in a blocking solution (5% normal donkey serum [NDS] in PBS with 0.2% Triton X‐100 [PBT]) for an hour. Primary antibodies including rabbit anti‐mCherry (1:1000, Abcam, RRID: ab167453), rabbit anti‐GFP (1:1000, Thermo Fisher Scientific, catalog #11122, RRID: AB_221569), sheep anti‐TH (1:1000, Merck Millipore; RRID: AB1542), rabbit anti‐cFos (1:1000, Santa Cruz Biotechnology Cat# sc‐52, RRID: AB_2106783) were used. NTS sections were incubated in primary antibodies at room temperature overnight. The following day, sections were washed in PBS and incubated in a secondary antibody solution consisting of Alexa Fluor 488 anti‐rabbit (1:500, Thermo Fisher; RRID: AB_2535792) and Alexa Fluor 594 anti‐sheep (1:500, Thermo Fisher; RRID: AB_2534083) in 5% NDS and PBT at room temperature. After 2 h, sections were washed, mounted onto slides and coverslipped with Fluoromount™ Aqueous Mounting Medium (Sigma Aldrich, United States). Brain sections were visualized under a fluorescence microscope at 10× magnification with a 488, 594 and 405 nm filter to image GFP, mCherry and FG respectively.

Neurons expressing cFos, TH, FG, and their colocalization were manually counted using CellSens software (Olympus, Japan). The number of colocalized neurons was expressed as a proportion of the total number of neurons expressing TH and as a proportion of the total FG neurons. The experimenter was blinded to treatment conditions.

### 2.10 Statistical analyses

Data were reported as mean ± standard error of the mean (SEM) and analyzed using IBM SPSS Statistics 26. For the anatomical tracing experiment, independent *t*‐tests were used to compare differences in the proportion of NTS neurons expressing FG, TH, and cFos between rats in the Deprived and Re‐fed groups. For behavioral experiments, a three‐way mixed design analysis of variance (ANOVA) was used where the within‐subject factors were treatment and time or cue and the between‐subject factor was genotype. When there was a significant interaction, analysis was split into genotype. For each genotype, repeated measures ANOVA was performed followed by pairwise comparisons with Bonferroni correction for multiple comparisons to mitigate potential Type I error (home cage chow intake, body weight) or paired *t*‐tests (cue‐induced appetitive behavior) to determine within‐subject differences between treatments. Results were considered statistically significant at *p* < .05.

## 3 RESULTS

### 3.1 A2^NTS^ ^→LH^ neurons are activated by food intake

**FIGURE 1:**
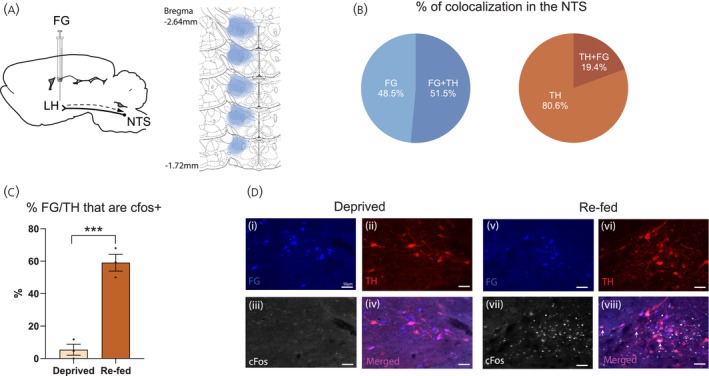
NTS A2 neurons project to the LH and are activated by food intake (A) Schematic of Fluorogold (FG) infusion to the LH and FG expression throughout the LH (B) Pie charts showing the percentage of colocalization between TH and FG neurons in the NTS. Left: 51.5% of FG‐expressing neurons also express TH. Right: 19.4% of TH neurons also express FG. (C) Re‐fed rats (*n* = 3) showed a higher percentage of cFos‐positive FG + TH neurons (A2^NTS→LH^) compared to Deprived rats (*n* = 3). (D) Representative images of the NTS from Deprived and Re‐fed rats showing neurons labelled for FG (i, v), TH (ii, vi), cFos (iii, vii) and their colocalization (iv, viii). Independent sample t‐test was conducted to determine differences between groups. Data are expressed as mean ± SEM. ***p < .001.

Anatomical tracing results showed that NTS A2 neurons project to the LH, where 51.5 ± 8.1% FG‐expressing A2 cells colocalize with TH and 19.4 ± 4.1% of TH‐expressing A2 cells also express FG (Figure 1A,B). To determine whether A2^NTS→LH^ neurons are activated by food intake, we quantified the number of activated A2^NTS→LH^ neurons in the Deprived and Re‐fed groups. We first showed that there were significantly more cFos‐expressing cells in the NTS in Re‐fed compared to the Deprived group (116.1 ± 16.2 vs. 5.4 ± 2.1; *t*_4_ = 6.791, *p* < .001), indicating that food intake activated NTS neurons. Further analysis on A2^NTS→LH^ neurons showed that there were more A2^NTS→LH^ neurons expressing cFos in the Re‐fed group compared to the Deprived group (*t*_4_ = 8.625, *p* < .001; Figure 1C,D). Together, these results provide initial evidence that NTS A2 neurons project to the LH and this projection is activated by food intake.

### 3.2 Chow intake

To determine whether A2^NTS→LH^ neurons are involved in food intake control, we performed chemogenetic activation of A2^NTS→LH^ neurons and examined their effects on chow intake. The assumption of sphericity was met for treatment (χ^2^(2) = 0.480, *p* = .787) but was violated for time (χ^2^(2) = 32.309, *p* < .001). Greenhouse–Geisser corrections were therefore applied for time (ε = 0.51). A three‐way ANOVA revealed a main effect of treatment (*F*_2,22_ = 5.312, *p* < .05), time (*F*_1.02,11.222_ = 53.455, *p* < .001), treatment × genotype (*F*_2,22_ = 7.461, *p* < .01), and treatment × time × genotype interaction (*F*_4,44_ = 4.832, *p* < .05). Analysis was subsequently split into genotype.

**FIGURE 2:**
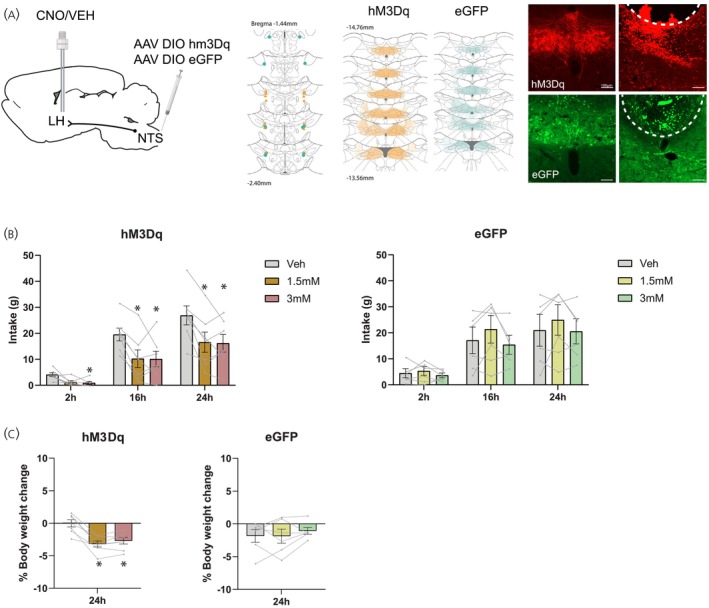
Chemogenetic activation of A2^NTS→LH^ neurons on chow intake. (A) Schematic of LH cannula implantation and viral infusions to the NTS. Placement maps for LH cannula (hM3Dq rats in orange triangles and eGFP rats in teal circles) and NTS hM3Dq and eGFP expression with representative images in the NTS and LH. Dotted lines show cannula tract. Some autofluorescence is visible at the injection site due to Pontamine Sky Blue infusions prior to perfusions. (B) Chow intake (g) at 2, 16 and 24 h after dark cycle onset in hM3Dq (*n* = 7) and eGFP (*n* = 6) rats following LH infusions of VEH, 1.5 mM or 3 mM CNO. (C) Percent changes in body weight of hM3Dq and eGFP rats following LH infusions of VEH, 1.5 mM or 3 mM CNO. Each line represents individual rats. Mixed model three‐way ANOVA was performed for all groups followed by repeated measures ANOVA and pairwise comparisons with Bonferroni correction for multiple comparisons to determine differences between treatments for each genotype. Data are expressed as mean ± SEM. *p < .05.

In rats injected with the excitatory DREADD hM3Dq (hM3Dq rats), the sphericity was also violated for time (χ^2^(2) = 13.784, *p* = .001) but not treatment (χ^2^(2) = 1.459, *p* = .482) or time × treatment (χ^2^(9) = 10.402, *p* = .352) and Greenhouse–Geisser corrections were applied for time (ε = 0.516). There was a main effect of treatment (*F*_2,12_ = 12.276, *p* < .01), time (*F*_1.033,6.197_ = 31.840, *p* < .001) and time × treatment interaction (*F*_4,24_ = 6.071, *p* < .01). Pairwise analysis with Bonferroni correction revealed that, compared to vehicle administration, 1.5 mM CNO reduced chow intake at 16 h (*p* < .01) and 24 h (*p* < .05) but not at 2 h (*p* = .06), and 3 mM CNO decreased chow intake at 2 h (*p* < .05), 16 h (*p* < .05) and 24 h (*p* < .05; Figure 2B).

For rats injected with the eGFP reporter control (eGFP rats), the assumption of sphericity was violated for time (χ^2^(2) = 17.119, *p* < .001) but not treatment (χ^2^(2) = 1.678, *p* = .432) or treatment × time (χ^2^(9) = 9.928, *p* = .411). Greenhouse–Geisser corrections were therefore applied for time (ε = .503). While there was a main effect of time (*F*_1.007,5.035_ = 22.609, *p* < .01), there was no effect of treatment (*F*_2,10_ = 1.340, *p* = .305) or treatment × time interaction (*F*_4,20_ = 1.093, *p* = .387), indicating that CNO had no effect on chow intake (Figure 2B) and demonstrating its specificity to rats expressing hM3Dq. Together, results showed that activation of A2^NTS→LH^ neurons reduced chow intake.

### 3.3 Body weight

We also examined the effects of A2^NTS→LH^ neuron activation on body weight. Mauchly's test of sphericity was not violated (χ^2^(2) = 0.937, *p* = .626). A two‐way ANOVA revealed a main effect of treatment (*F*_2,22_ = 6.111, *p* < .01) and treatment × genotype interaction (*F*_2,22_ = 8.561, *p* < .01). When analysis was split into genotype, results showed that in hM3Dq rats, the assumption of sphericity was met (χ^2^(2) = 3.310, *p* = .191) and there was a main effect of treatment (*F*_2,12_ = 31.19, *p* < .001) where both 1.5 and 3 mM CNO significantly reduced body weight 24 h after injections (*p* < .01; Figure 2C). In eGFP rats, the assumption of sphericity was also met (χ^2^(2) = 0.114, *p* = .945) and there was no effect of treatment (*F*_2,10_ = 0.567, *p* = .585) on body weight (Figure 2C). Thus, activation of A2^NTS→LH^ neurons also suppressed body weight.

### 3.4 Cue‐induced appetitive behavior

**FIGURE 3:**
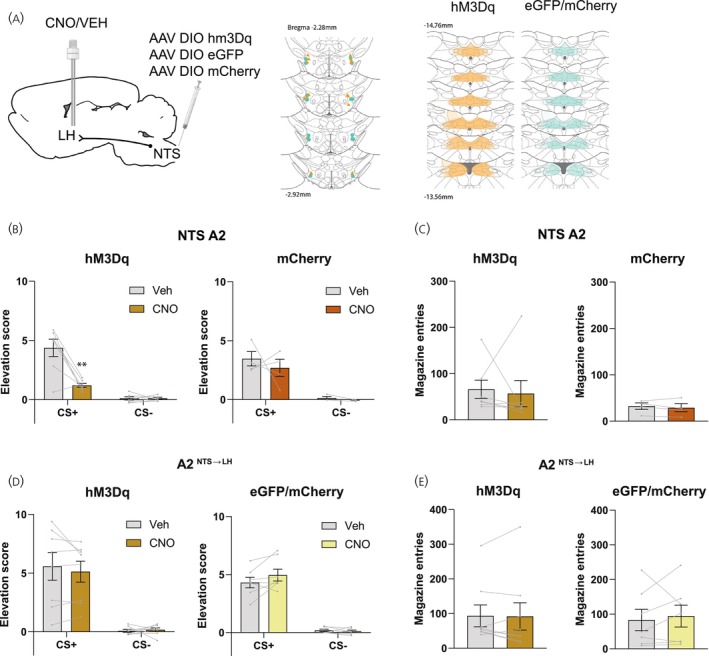
Chemogenetic activation of NTS A2 and A2^NTS→LH^ on cue‐induced appetitive behaviors. (A) Schematic of LH cannula implantation and viral infusions to the NTS. Placement maps for LH cannula (hM3Dq rats in orange triangles and eGFP/mCherry rats in teal circles), NTS hM3Dq and eGFP/mCherry expression. (B) Elevation scores and (C) magazine entries of hM3Dq (*n* = 8) and eGFP/mCherry (*n* = 7) rats following i.p. injection of CNO to stimulate NTS A2 neurons. (D) Elevation scores and (E) magazine entries of hM3Dq and eGFP/mCherry rats following LH infusions of VEH or 3 mM CNO to stimulate A2^NTS→LH^ neurons. Each line represents individual rats. Mixed model three‐way ANOVA was performed for all groups followed by repeated measures ANOVA and paired *t*‐tests to determine differences between treatments for each genotype. Data are expressed as mean ± SEM. **p < .01.

We first examined whether NTS A2 neurons are involved in non‐homeostatic food intake control by assessing cue‐induced appetitive behavior using the Pavlovian appetitive conditioning task. TH Cre rats received i.p. injections of CNO or VEH to activate all NTS A2 neurons. To assess cue‐induced appetitive behavior, we calculated elevation scores, defined as the number of magazine entries during the 15 s CS± period minus the number of entries during the pre CS± period (i.e., 15 s before CS onset). Analysis of the elevation scores revealed a main effect of treatment (*F*_1,9_ = 11.170, *p* = .009) and CS (*F*_1,9_ = 153.660, *p* < .001) but not genotype (*F*_1,9_ = 0.096, *p* = .763). Whilst there was no CS × genotype (*F*_1,9_ = 0.618, *p* = .452) or treatment × genotype (*F*_1,9_ = 3.658, *p* = .088) interaction, there was a CS × treatment (*F*_1,9_ = 13.327, *p* = .005) and genotype × treatment × CS interaction (*F*_1,9_ = 6.400, *p* = .032). Data were then split by genotype, and repeated measures ANOVA was performed. In hM3Dq group, there was a significant main effect of CS (*F*_1,6_ = 66.552, *p* < .001), treatment (*F*_1,6_ = 32.130, *p* = .001), and CS × treatment (*F*_1,6_ = 47.111, *p* < .001) interaction. Further paired *t*‐test analyses were conducted to compare the effect of VEH vs. CNO treatment on CS elevation scores. In hM3Dq rats, paired *t*‐test analysis showed that CNO but not VEH, significantly suppressed CS+ (*t*_6_ = 6.424, *p* < .01) elevation scores, but not CS− (*t*_6_ = 0.000, *p* = 1.00). In the mCherry rats, there was a significant main effect of CS (*F*_1,3_ = 317.157, *p* < .001) but not treatment (*F*_1,3_ = 0.441, *p* = .554) or CS × treatment (*F*_1,3_ = 0.262, *p* = .644) interaction (Figure 3B), indicating that whilst mCherry rats learnt the task, CNO had no effect on cue‐induced appetitive behavior. When inter‐trial interval magazine entries were analyzed, analysis showed no main effect of treatment (*F*_1,9_ = 0.109, *p* = .749) or treatment × genotype (*F*_1,9_ = 0.027, *p* = .873) interaction (Figure 3C). This indicates that magazine entries did not differ between mCherry and hM3Dq groups when administered VEH or CNO. Together, these results show that activation of all NTS A2 neurons suppressed cue‐induced appetitive behavior in response to CS+, without affecting CS− elevation scores or intertrial interval magazine entries.

We next investigated whether activation of A2^NTS→LH^ neurons suppresses cue‐induced appetitive behavior. Given that there were no differences in CNO doses on home cage chow intake, only 3 mM CNO was used in this experiment. TH Cre rats received microinjections of CNO or VEH to the LH to selectively target the A2^NTS→LH^ neurons. A three‐way mixed ANOVA revealed a main effect of CS (*F*_1,13_ = 71.933, *p* < .001), where elevation was higher in CS+ compared to CS−, suggesting that all rats exhibited a conditioned response towards CS+ but not CS−. However, there was no treatment × genotype × CS interaction (*F*_1,13_ = 3.769, *p* = .074), suggesting that CNO treatment had no effect on elevation scores for CS+ and CS− between hM3Dq and eGFP/mCherry control rats (Figure 3D). Similarly, with magazine entries, there was no genotype × treatment interaction (*F*_1,13_ = 0.289, *p* = .600; Figure 3E). Taken together, chemogenetic activation of the A2^NTS→LH^ pathway had no effect on cue‐induced appetitive behaviors.

## 4 DISCUSSION

This study examined the role of A2^NTS→LH^ neurons in regulating both homeostatic and non‐homeostatic feeding behaviors. Using anatomical tracing and pathway‐specific chemogenetics, we showed for the first time that NTS A2 neurons projecting to the LH are activated by food intake and that activating A2^NTS→LH^ pathway suppressed homeostatic food intake and body weight but did not affect cue‐induced appetitive behavior. In contrast, broader activation of NTS A2 neurons did suppress cue‐driven feeding. These findings provide the first evidence that the A2^NTS→LH^ pathway selectively participates in the regulation of homeostatic but not non‐homeostatic cue‐induced feeding behaviors, indicating functional segregation within NTS A2 neural circuits that differentially modulate distinct aspects of feeding behaviors.

### 4.1 NTS A2 neurons projects to the LH to control food intake

We identified the LH as a novel downstream target of NTS A2 noradrenergic neurons that contributes to food intake control. Our findings showed that the A2^NTS→LH^ pathway is sensitive to re‐feeding, indicating that these neurons are triggered by food intake. While previous studies have shown that NTS A2 neurons are activated by gut signals and food intake, our study specifically highlights the recruitment of the A2^NTS→LH^ pathway.^10^, ^28^, ^29^, ^30^ Supporting the hypothesis that A2^NTS→LH^ neurons are involved in food intake control, we showed that chemogenetic activation of A2^NTS→LH^ neurons significantly reduced chow intake and body weight over 24 h. This is in line with the intake inhibitory role of NTS A2 neurons and extends the intake inhibitory NTS A2 neural circuits (i.e., projections to the PVH and the parabrachial nucleus (PBN)) to also include the A2^NTS→LH^ pathway.^6^, ^28^ While NTS A2 projections to the PBN do not collateralize with forebrain regions, some collateralization exists between projections to the LH and PVH.^31^, ^32^ This suggests that the intake inhibitory effects observed with A2^NTS→LH^ neuron activation could be influenced by PVH collaterals. We think that this is unlikely since CNO was microinjected to the LH which minimizes antidromic effects. Nonetheless, NTS A2 collateralization to LH and PVH suggests that simultaneous activation of both these pathways could produce additive or complementary effects on intake suppression. These possibilities warrant further investigation.

Given that NTS A2 neurons express noradrenaline, it is possible that the observed intake inhibitory effects are mediated via increased noradrenaline release in the LH. Supporting this possibility is evidence from previous studies showing that LH microinjections of noradrenaline blunt feeding after food deprivation, indicating that LH noradrenaline signaling suppresses food intake.^33^ In addition to the NTS, another source of noradrenaline is the locus coeruleus (LC).^34^, ^35^ Activation of LC noradrenergic neurons also suppresses food intake by reducing meal number but not meal size.^36^, ^37^ Furthermore, LC neural activity increases during approach and declines during food intake, which suggests that LC noradrenergic‐induced intake suppression is mediated through the inhibition of meal initiation. NTS A2 neurons, on the other hand, are activated by food intake and are likely suppressing food intake through satiation/meal termination.^10^, ^28^, ^29^, ^30^ Thus, while both populations of noradrenergic neurons suppress food intake, they do so via distinct mechanisms. Together, findings from our study support the role of noradrenergic signaling in food intake control and further demonstrate a functional neural connection between the NTS A2 and LH neurons in regulating homeostatic food intake and body weight.

### 4.2 NTS A2 neuron activation reduces cue‐induced appetitive behaviors, independent of projections to the LH

LH neurons have also been implicated in non‐homeostatic feeding including cue‐induced feeding behaviors but whether this involves input from NTS A2 neurons was unclear. Using chemogenetics, we showed that activation of NTS A2 neurons broadly suppressed cue‐induced appetitive behavior, consistent with our previous findings.^38^ Given that LH neurons are activated by food‐associated cues and that stimulation of the ventral noradrenergic bundle (which includes NTS A2 neuron projections) inhibits LH activity via α‐adrenergic receptor signaling, we hypothesized that activation of A2^NTS→LH^ neurons would suppress cue‐evoked food approach behaviors.^21^, ^39^, ^40^, ^41^ However, contrary to our hypothesis, stimulation of A2^NTS→LH^ neurons had no effect on cue‐induced appetitive behavior. This indicates that this pathway alone is insufficient to suppress the ability of food cue to elicit conditioned approach. It is possible that suppression of cue‐driven appetitive behaviors requires other downstream targets or coordinated activity across multiple projections from the NTS A2 neurons.

One possible candidate is the NAc, which receives noradrenaline input primarily from NTS A2 neurons.^42^ Our recent findings showed that noradrenaline in the NAc is reduced during food cue presentation and cue‐induced approach, suggesting that noradrenaline levels are dampened to promote cue‐induced approach.^10^ Therefore, augmenting noradrenaline levels would lead to behavioral suppression, which is consistent with our results here that global activation of NTS A2 neurons suppresses cue‐induced approach. Another possibility is via their projection to the paraventricular thalamus (PVT). We previously showed that the PVT regulates cue‐induced reinstatement of food‐seeking behavior, and noradrenergic signaling in the PVT has been shown to regulate feeding behaviors.^22^, ^43^ Thus, it is possible that NTS A2 neurons affect cue‐induced appetitive behaviors via their projections to PVT. Future studies would need to explore these possible pathways to better elucidate the NTS A2 neural circuits contributing to cue‐induced feeding behaviors.

Given that 50% of NTS neurons that project to the LH are not A2 neurons, it is possible that other NTS inputs to the LH may contribute to food cue processing. Notably, distinct from A2 neurons are the preproglucagon neurons and they project to the LH.^44^, ^45^ While their role in cue‐driven feeding behaviors is still unknown, GLP‐1 receptors in the LH are involved in the control of food intake and food‐motivated behavior.^14^ Whether other populations of NTS neurons project to the LH remains to be determined.

Although we used cue‐induced appetitive behavior as one model of non‐homeostatic feeding behavior, we acknowledge that there are other aspects of non‐homeostatic feeding behavior such as cue‐induced motivation and food‐seeking that were not assessed in the present study. Thus, future studies could examine how NTS A2 neurons regulate other non‐homeostatic feeding behaviors using behavioral assays such as Pavlovian instrumental transfer or conditioned place preference to better understand the role of NTS A2 neurons and their downstream projections in regulating non‐homeostatic feeding behaviors.

### 4.3 Possible mechanism mediating the effects of A2^NTS^ ^→LH^ neurons on homeostatic but not non‐homeostatic feeding behaviors

The selective participation of A2^NTS→LH^ neurons in homeostatic feeding but not in cue‐induced appetitive behaviors suggests that the NTS A2 inputs may target LH neurons that are specifically involved in homeostatic feeding, rather than those mediating cue‐driven responses. The LH is a highly heterogeneous structure with diverse neurochemical populations that contribute to various aspects of feeding behaviors.^46^ Interestingly, many LH neuron populations involved in homeostatic feeding also contribute to cue‐induced feeding. For example, inhibition of LH GABAergic neurons not only decreases homeostatic food intake but also reduces the acquisition and expression of Pavlovian conditioning to a food reward.^15^, ^19^ Similarly, two other populations of LH neurons – the melanin‐concentrating hormone (MCH) and orexin‐expressing neurons – stimulate food intake and are also involved in cue‐induced feeding. Both populations of LH neurons are activated by food‐associated cues and contexts, and stimulation of MCH neurons increases cue‐ and context‐induced appetitive food‐seeking behaviors while inhibition of orexin receptors suppresses cue‐induced feeding.^21^, ^47^, ^48^, ^49^, ^50^ These findings therefore suggest that LH GABAergic, MCH, and orexin neurons may be involved in regulating feeding behaviors driven by both homeostatic and non‐homeostatic signals. Given that activation of NTS A2 neurons that project to the LH only affects homeostatic food intake but not cue‐induced appetitive behaviors, it is unlikely that the aforementioned LH neuron populations mediate the effects of A2^NTS→LH^ stimulation.

There are other LH neuron populations that mediate different feeding behaviors. For example, LH galanin neurons do not directly affect food intake but they enhance food‐motivated behavior.^51^ Similarly, LH neurotensin neurons reduce food intake when stimulated but are not activated by the auditory cue that predicts food reward. Instead, they are recruited during head entries to retrieve the food reward.^52^ This suggests that LH neurotensin neurons are sensitive to consummatory signals but not to food‐predictive cues. Further supporting this distinction, inactivation of neurotensin receptor signaling does not impair the acquisition of the cue‐based operant task but increases latency to enter the food magazine towards the end of the session, suggesting a role for these neurons in regulating satiation, rather than cue‐driven feeding.^52^ Importantly, LH neurotensin neurons receive monosynaptic input from the NTS, raising the possibility that NTS A2 neurons modulate feeding behaviors via LH neurotensin neurons.^53^ Given the selective involvement of LH neurotensin neurons in homeostatic food intake, but not in cue‐driven feeding behaviors, it is tempting to speculate that the differential effects of A2^NTS→LH^ stimulation on home cage food intake and cue‐induced appetitive behavior could be mediated via NTS A2 input to LH neurotensin neurons. Future studies are warranted to test this hypothesis and identify LH neural subtypes that receive synaptic input from NTS A2 neurons to determine the mechanism underlying the divergent effects of A2^NTS→LH^ stimulation on homeostatic food intake and cue‐induced appetitive behavior.

In summary, our findings demonstrate that NTS A2 neurons regulate homeostatic feeding and cue‐induced feeding via distinct neural mechanisms. Specifically, A2^NTS→LH^ neurons contribute to homeostatic food intake, but not cue‐induced feeding behaviors. This work highlights the functional diversity of LH neurons and the need to dissect the LH neural phenotypes and NTS neural circuits to better understand the neural mechanisms underlying different feeding behaviors.

## AUTHOR CONTRIBUTIONS

**Jo Ann Yap:** Investigation; data curation; methodology; formal analysis; writing – original draft; writing – review and editing. **Helen Yi Sha Gu:** Methodology; investigation; formal analysis; writing – review and editing. **Colin Vuong:** Conceptualization; methodology; investigation; formal analysis; validation; writing – original draft; writing – review and editing; data curation. **Hun Yee Lai:** Writing – review and editing; methodology; investigation; formal analysis. **Zhi Yi Ong:** Conceptualization; methodology; data curation; supervision; formal analysis; investigation; funding acquisition; writing – review and editing; writing – original draft; project administration.

## CONFLICT OF INTEREST STATEMENT

The authors declare no conflict of interest.

## Data Availability

The data that support the findings of this study are available from the corresponding author upon reasonable request.
